# Discovery of Novel Small Molecules that Block Myofibroblast Formation: Implications for Capsular Contracture Treatment

**DOI:** 10.1097/GOX.0000000000002430

**Published:** 2019-09-30

**Authors:** Rachel H. Park, Stephen J. Pollock, Richard P. Phipps, Howard N. Langstein, Collynn F. Woeller

**Affiliations:** From the * University of Rochester School of Medicine and Dentistry, Rochester, N.Y.; † Department of Environmental Medicine, University of Rochester Medical Center, Rochester, N.Y.; ‡ Division of Plastic and Reconstructive Surgery, University of Rochester Medical Center, Rochester, N.Y.; § Department of Ophthalmology, University of Rochester School of Medicine and Dentistry, Rochester, N.Y.

## Abstract

Capsular contracture is a devastating complication that occurs in patients undergoing implant-based breast reconstruction. Ionizing radiation drives and exacerbates capsular contracture in part by activating cytokines, including transforming growth factor-beta (TGF-β). TGF-β promotes myofibroblast differentiation and proliferation, leading to excessive contractile scar formation. Therefore, targeting the TGF-β pathway may attenuate capsular contracture.

**Methods:**

A 20,000 small molecule library was screened for anti-TGF-β activity. Structurally diverse anti-TGF-β agents were identified and then tested on primary human capsular fibroblasts. Fibroblasts were irradiated or not, and then treated with both TGF-β and candidate molecules. Resulting cells were then analyzed for myofibroblast activity using myofibroblast markers including alpha-smooth muscle actin, collagen I, Thy1, and periostin, using Western Blot, quantitative real-time polymerase chain reaction, and immunofluorescence.

**Results:**

Human capsular fibroblasts treated with TGF-β showed a significant increase in alpha-smooth muscle actin, collagen I, and periostin levels (protein and/or mRNA). Interestingly, fibroblasts treated with latent TGF-β and 10 Gy radiation also showed significantly increased levels of myofibroblast markers. Cells that were treated with the novel small molecules showed a significant reduction in myofibroblast activation, even in the presence of radiation.

**Conclusions:**

Several novel small molecules with anti-TGF-β activity can effectively prevent human capsular fibroblast to myofibroblast differentiation in vitro, even in the presence of radiation. These results highlight novel therapeutic options that may be utilized in the future to prevent radiation-induced capsular contracture.

## INTRODUCTION

Breast cancer is the most common malignancy and a second most common cause of cancer deaths in US women, with the American Cancer Society estimating more than 266,000 new cases of invasive breast cancer diagnosis in 2018.^1^ The mainstay of breast cancer treatment is surgical resection, both lumpectomy and mastectomy, often followed by radiation therapy.^2^ Although the aforementioned treatments have helped to significantly decrease the mortality rate, many patients must face the consequences of body disfiguring surgery. Consequently, there have been a gradually increasing number of breast cancer patients who chose to undergo breast reconstructions after mastectomy. Albornoz et al.^3^ show that the rate of immediate breast reconstruction has steadily increased about 5% per year, resulting in a 78% increase from 1998 to 2008. The 2 main methods of breast reconstruction surgery are autologous and implant-based reconstruction, with autologous procedure being the traditionally more popular option. However, there has been a paradigm shift in recent years, as the rate of implant-based reconstruction after mastectomy surpassed that of autologous reconstruction around 2002, and continues to be the more popular procedure.^3^

Unfortunately, many of the patients who receive implant-based breast reconstruction, and especially those who receive adjuvant radiation therapy, suffer from a number of complications, capsular contracture being the most common long-term complication.^4^ Capsular contracture is a painful, debilitating condition in which excessive scar tissue encapsulates the implant, causing chronic stiffness and disfigurement.^5^ There are many factors that can increase the risk of capsular contracture, such as periprosthetic infection, foreign response to implant material, and location of the implant.^6,7^ Ionizing radiation, while effective in killing residual malignant cells, is one of the most significant factors that increases capsular contracture rate, up to 7-fold.^8^ Currently, it is unclear how ionizing radiation leads to the development of excessive and pathologic fibrosis. One possible mechanism is that radiation treatment leads to inflammatory cytokine cascades, which cause abnormal wound healing.^9^ Among those cytokines, transforming growth factor-beta (TGF-β), a prowound healing molecule, stands out as a crucial player that acts as a “master switch” to activate the fibrosis pathway.^9^

TGF-β is a member of a family of cellular mediators that are crucial in tissue homeostasis, cellular regulation and growth, wound healing and tissue repair, and some suggest it has a role in tumor initiation and progression.^10,11^ There are 3 different isoforms of TGF-β, which are TGF-β1, β2, and β3, with TGF-β1 being the most prevalent isoform associated with tissue fibrosis.^10,12^ In early wound healing, activation of TGF-β1 promotes endothelial cell proliferation, extracellular matrix (ECM) production, and angiogenesis.^13^ In later phases, TGF-β1 promotes myofibroblast differentiation, activation, and proliferation.^10,14^ Myofibroblast activation leads to wound contracture and closure, as these cells produce ECM and promote contracture caused by prominent alpha-smooth muscle actin (αSMA) containing stress fibers.^15^ Animal models show organ and tissue fibrosis following exogenous TGF-β1 injections or with genetic TGF-β1 overexpression, hence demonstrating its causative role in fibrosis.^16,17^

Currently, there is a major unmet need for safe, effective, and widely available treatments that can target fibrotic diseases, radiation-induced capsular contracture being one of them. Given that TGF-β is one of the most powerful players in driving fibrosis, attenuating excessive TGF-β signaling may provide an effective treatment strategy for capsular contracture, and potentially for other fibrotic diseases as well.

The primary goal of this study is to identify novel small molecules with anti-TGF-β activity that can effectively prevent myofibroblast formation in human breast capsular fibroblasts. Our previous study identified salinomycin as a powerful antiscarring agent, which effectively blocked TGF-β–driven fibroblast proliferation and myofibroblast formation in vitro.^18^ Salinomycin is a polyether ionophore that has been approved for coccidiosis control and promotion of animal growth and feed efficiency.^19^ Although this previous study showed that salinomycin possesses anti-TGF-β activity and reduced myofibroblast activity in vitro, there are needs to find additional therapeutic small molecule drugs which may be easier to develop and synthesize. Salinomycin is a natural product with a high molecular weight that has potent Na+ ionophore activity, which makes it a challenging target for structure modification for optimizing desired activity. Furthermore, it is produced by fermentation, which is costly to maintain on a large scale. Alternatively, chemically synthesized small molecules may provide well-defined structures that can be modified to optimize the desired anti-TGF-β activity and can potentially prevent excessive fibrosis and scarring. Here, we report a new class of molecules that potently block TGF-β1 activity and capsular myofibroblast differentiation.

## METHODS

### Cell Culture

Primary fibroblast strains were established from deidentified breast skin and peri-implant capsular tissue explants from several different individuals. All samples were handled appropriately following authorization from the University of Rochester IRB. All the capsular fibroblast strains used in this study were harvested from individuals without a history of radiation therapy. Tissue samples were minced and cultured in Dulbecco’s Modified Eagle Medium (DMEM) supplemented with 10% fetal bovine serum (FBS, HyClone Labs, Logan, Utah) and 1% Antibiotic-Antimycotic solution (A/A, ThermoFischer Scientific, Waltham, Mass.). Cells were expanded onto culture dishes and maintained in complete growth medium (DMEM + 10% FBS + 1% A/A); resulting cells were morphologically consistent with fibroblasts and expressed mesodermal markers such as vimentin and Thy1 (CD90). Capsular fibroblasts were seeded onto 6-well plates and cultured until reaching 60%–80% confluence, then washed with 1× Phosphate-Buffered Saline (PBS, Gibco, Carlsbad, Calif.), and cultured in reduced serum media (RSM; DMEM + 0.05% FBS+ 1% A/A) for 48 hours. After serum reduction, cells were treated in fresh RSM containing 2.5 ng/mL TGF-β1 (R&D Systems, Minneapolis, Minn.) with or without varying concentrations of novel small molecules.

### Small Molecule Screen

To identify compounds that inhibit TGF-β activity and thus may be novel antiscarring compounds, the ChemBridge diversity set of 20,000 small molecules was screened using a previously established assay.^18^ Briefly, the TGF-β–responsive HEK293FT-SBE-Tk-luc cell line was used for the screening.^18^ This initial screening revealed 80 small molecules with TGF-β inhibition ranging from 44% to 99% (based on the control TGF-β receptor inhibitor, SB-431552).^20^ After library screening, additional compound was purchased from Chembridge Corporation (San Diego, Calif.). These 80 compounds were subsequently tested on primary human capsular fibroblasts, during which the cells were treated with TGF-β1 (2.5 ng/mL) to induce myofibroblast differentiation and with various doses of small molecule to test for their anti-TGF-β activity. Samples were analyzed by probing for markers of myofibroblast activity as described below, αSMA being one of the most prominent and well-established markers of myofibroblasts.^21^ Collagen I levels were also measured to further assess myofibroblast activity, which followed a similar pattern of αSMA expression. In addition, Thy1 (formally called CD90) levels were also analyzed.

### Western Blotting

Total cellular protein was isolated and analyzed by Western blot as described previously.^22^ Primary antibodies used in this study include αSMA (1:1,000, mouse, Sigma-Aldrich, St. Louis, Mo.) and Thy 1 (1:500, sheep, R&D Systems, Minneapolis, Minn.). Secondary antibodies, goat antimouse (1:20,000) and donkey antisheep (1:5,000), conjugated to horseradish peroxidase were used (Jackson Immunoresearch, West Grove, Pa.).

### Collagen Production Assay

Cell culture supernatant was collected and transferred (10 µL diluted in 90 µL 1× PBS) to a polyvinylidene difluoride (PVDF) membrane using a slot blot device (PR 648 Slot Blot Manifold, GE Healthcare Bio-Sciences, Pittsburgh, Pa.) and vacuum pump. The membrane was incubated in 5% nonfat dry milk in 1× PBS to block nonspecific protein binding, then probed with antitype I collagen antibody (goat, 1:4,000, Santa Cruz Biotechnology, Dallas, Tex.), washed with 1× PBS containing 0.1% Tween-20, then incubated with horseradish peroxidase (HRP)-conjugated secondary antibody (donkey antigoat, 1:5,000, Santa Cruz Biotechnology). Band intensities were quantified using Image Lab software, and values were normalized to vehicle treatments.

### Quantitative Real-time Polymerase Chain Reaction

Total cellular RNA was extracted using a Qiazol lysis reagent and isolated using RNeasy Mini Kit (Qiagen, Valencia, Calif.) according to the manufacturers’ instructions. Total RNA concentrations were determined with a NanoDrop 1000 spectrophotometer (Thermo Scientific, Wilmington, Del.). Fifty nanograms of RNA per sample was used to generate cDNA with iScript reverse transcriptase (Bio-Rad, Hercules, Calif.). Real-Time Polymerase Chain Reaction (RT-PCR) was performed using SSoFast SYBR Green PCR master mix reagent (BioRad) and an iCycler iQ5 PCR thermal cycler to measure relative mRNA levels of Thy1, Col1A, and periostin (Postn); expression was normalized to the levels of 18S rRNA in the corresponding samples. Gene-specific primers were as follows: ACTA2 [αSMA; 5′-CCCACAATGTCCCCATCT-ATG-3′ (forward) and 5′-AGTTTCTCCTTGATGTCCCG-3′ (reverse)]; 18S rRNA [18S; 5′-TGAGAAACGGCTACCACATC-3′ (forward) and 5′-ACTACGAGCTTTTTAACTGC-3′]; collagen 1A [Col1A; 5′-CCCCTGGAAAGAATGGAGATG-3′ (forward) and 5′-TCCAAACCACTGAAACCTCTG-3′ (reverse)]; Thy1 [Thy1; 5′-ATCTCCTCCCAGAACGTC-3′ (forward) and 5′-ATCTCTGCACTGGAACTTG-3′ (reverse)]; periostin [Postn; 5′-CAACGGGCAAATACTGGAAAC-3′ (forward) and 5′-TCTCGCGGAATATGTGAATCG-3′ (reverse)].

### Immunofluorescence

Cultured and treated primary capsular fibroblasts were fixed using 100% methanol fix for 15 minutes. After fixing, cells were rinsed in 1× PBS. Cells were then blocked using 5% BSA in 1× PBS for 1 hour with gentle rocking at room temperature. Cells were incubated with primary αSMA antibody (rabbit, 1:1,000, Abcam, Cambridge, UK) for 1 hour at room temperature (RT). After primary incubation, antibody solution was removed and washed with 1× PBS 3 times. Cells were then incubated with secondary antibody (AlexaFluor 647 conjugated goat antirabbit, 1:500, ThermoScientific, Waltham, MA) for 45 minutes at RT. The secondary solution was removed and then washed 3 times with 1× PBS before visualizing with an EVOS cell imaging system (Invitrogen, Carlsbad, CA).

### Irradiation

Primary human capsular fibroblasts were cultured and serum reduced as described above. RSM was discarded and replaced with either fresh RSM or complete growth medium. Cells were treated with either 2.5 ng/mL latent TGF-β1 or 2.5 ng/mL of active TGF-β, with or without ChemB-737 (250 nM). Cells were irradiated using a 137 Cesium gamma ray source at approximately 2.73 cGy/min dose rate at 10 Gy, then incubated for 120 hours until harvest.

### Statistical Analysis

Data were analyzed using GraphPad Prism (Version 7) utilizing one-way analyses of variance with Sidak’s correction for multiple comparisons. *P* values <0.05 were considered significant.

## RESULTS

**Fig. 1.**
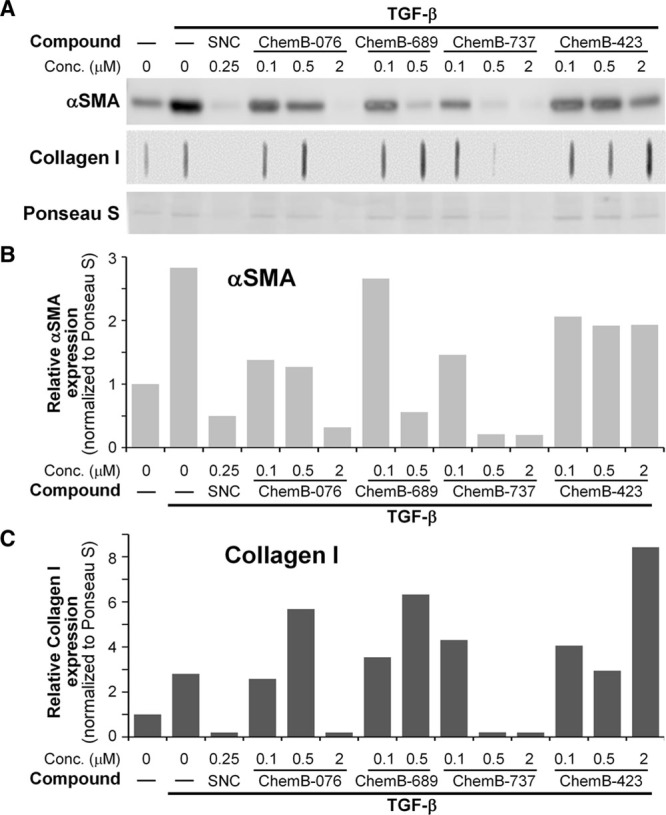
Newly identified small molecules block myofibroblast formation in human capsular fibroblasts. Western blot and slot-blot results of TGF-β1–treated human breast capsular fibroblasts treated with 4 different small molecules (time point = 72 hours). Levels of αSMA and collagen I, which are well-known myofibroblast markers, were measured. A, Representative blot images of αSMA and collagen I levels and Ponceau S stain (a total protein stain) for normalization. B and C, Quantification of αSMA and collagen I levels. Data were normalized to the control vehicle group (lane 1, no TGF-β nor treatment). TGF-β1 (2.5 ng/mL, final concentration) alone (lane 2) shows significant increase in myofibroblast marker levels compared with that of the vehicle group (lane 1). As a positive control for antimyofibroblast activity, some cells were cotreated with salinomycin (0.25 µM in lane 3). Some cells were also cotreated with ChemB-076, ChemB-689, ChemB-737, or ChemB-423 at the doses listed. Although all ChemB compounds tested show reductions in αSMA, only ChemB-737 shows significant reductions in both αSMA and collagen. SNC indicates salinomycin.

Radiation-induced capsular contracture stems from excessive and prolonged myofibroblast proliferation and activity, which is responsible for excessive ECM and collagen production, and αSMA expression that leads to contracture.^10,23^ Ionizing radiation can lead to activation and prolonged expression of TGF-β, which can lead to pathologic fibrosis and scarring, one such condition being radiation-induced capsular contracture.^10,24^ Hence, we hypothesized that blocking TGF-β activation would lead to reduced scarring and fibrosis, and potentially prevent radiation-induced capsular contracture. To find a novel small molecule that can effectively inhibit TGF-β activity, we screened a small molecule library that included 20,000 small molecules. Initial screening revealed 80 potential novel TGF-β inhibitors that were then tested in primary human capsular fibroblasts treated with TGF-β1 to induce myofibroblast formation. As expected, cells treated with TGF-β1 led to a robust myofibroblast phenotype, shown by αSMA expression and collagen 1A1 production, ranging from 2- to 10-fold above that of vehicle-treated cells (Fig. 1).

**Fig. 2.**
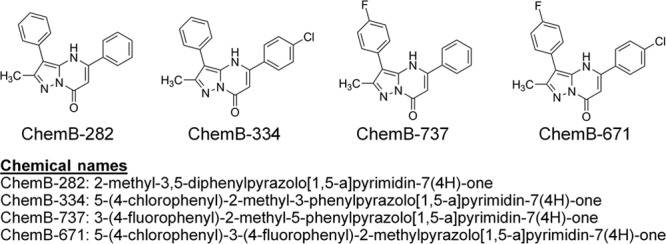
Molecular structures and chemical names of ChemB-737 and its analogs. These compounds showed potent anti–transforming growth factor-beta activity in primary human capsular fibroblasts. Structural differences come from the absence and presence of halogens (fluorine and chlorine).

**Fig. 3.**
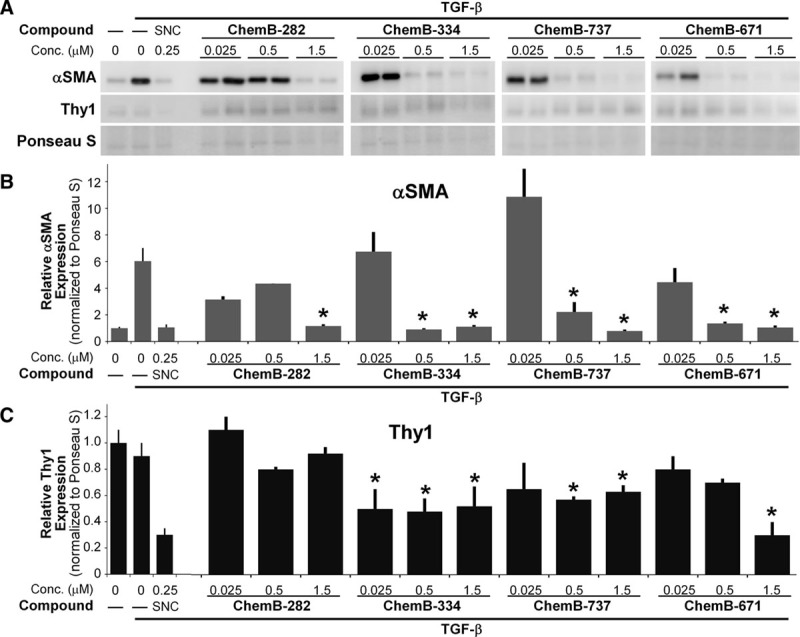
ChemB-282, 334, 737, and 671 effectively reduces both αSMA and Thy1 protein levels in TGF-β1-induced human breast capsular myofibroblasts. A, Representative blot images of αSMA and Thy1 levels and Ponceau S stain (a total protein stain) for normalization. B, Cells treated with TGF-β1 (2.5 ng/mL) without additional treatment led to a 6-fold increase in αSMA level, whereas cells treated with small molecules showed significantly reduced αSMA levels similar to that of the vehicle group. Molecules ChemB-334, 737, and 671 show significant reductions in αSMA levels at doses lower than 0.5 µM. C, Cells treated with novel small molecules ChemB-334, 737, and 671 led to significant reductions in Thy1 levels in the presence of TGF-β1. As a positive control for antimyofibroblast activity, some cells were cotreated with salinomycin (0.25 µM). The experiment was done in 3 times in triplicate wells and significance was tested using one-way analysis of variance with n = *P < 0.05. SNC indicates salinomycin.

Several of the small molecular inhibitors showed a dose-dependent reduction in myofibroblast formation (Fig. 1). One small molecule that stood out as a potent anti-TGF-β agent was molecule ChemB-737. ChemB-737 treatment at 500 nM showed a significant αSMA and collagen I reduction of about 80% compared with that of vehicle. Other small molecules, such as molecule ChemB-076 and ChemB-689, showed αSMA reduction as well but required significantly higher doses or reduced αSMA without reducing collagen production. ChemB-737 showed anti-TGF-β activity comparable to salinomycin,^18^ which we used as a control antimyofibroblast agent in these experiments. ChemB-737 also showed collagen I reduction to a level below that of vehicle-treated samples. Subsequently, we tested a total of 8 structural analogs of ChemB-737 (Fig. 2). Interestingly, we found that although small molecules ChemB-282, 334, 737, and 671 worked, 5 other analogs did not show any antimyofibroblast activity (Fig. 3).

**Fig. 4.**
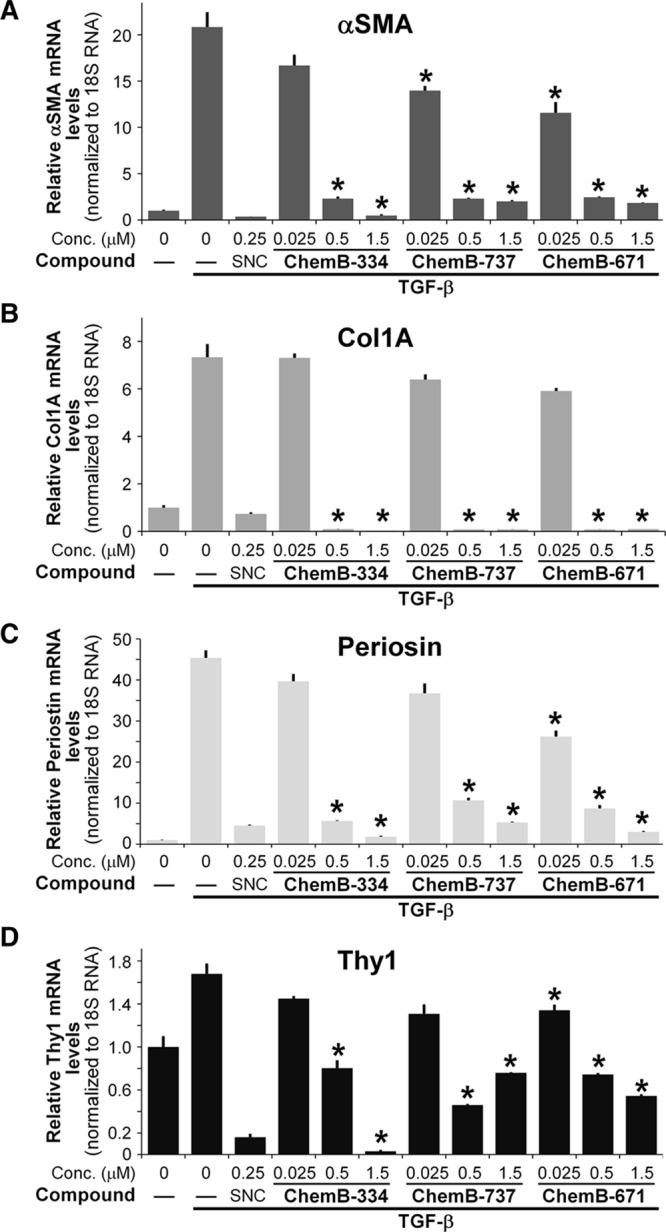
ChemB-334, 737, and 671 lead to significantly reduced mRNA levels of myofibroblast markers. αSMA (A), Col1A (B), Periostin (C), and Thy1 (D) in a dose-responsive manner in the presence of TGF-β. Cells that were exposed to TGF-β with only vehicle treatment (DMSO, Dimethyl Sulfoxide) showed significant increase in expression of these marker levels, such as a 20-fold increase in αSMA levels, 7-fold increase in collagen 1A levels, and an over 40-fold increase in periostin levels, indicating robust TGF-β–induced myofibroblast differentiation. Cells treated with ChemB-334, ChemB-737, or ChemB-671 show a dose-dependent decrease in expression of all myofibroblast markers. Importantly, the experiment was done 3 times in triplicate and significance was tested using one-way analysis of variance with n = *P < 0.05. SNC indicates xxx.

Gene expression of αSMA, collagen I, periostin, and Thy1 levels were analyzed using quantitative RT-PCR (qRT-PCR) to further test the antimyofibroblast activity of these molecules. We previously showed that Thy1 mRNA and protein expression were significantly increased in irradiated human peri-implant scar tissues.^25^ Furthermore, it was also shown that depletion of Thy1 led to less myofibroblast morphology and reduced αSMA level. Periostin is a secreted cell adhesion protein that plays a key role in wound healing and is another marker of myofibroblasts.^26^ TGF-β1 treatment led to a significant increase in expression of all of these markers indicating that fibroblasts readily differentiate into myofibroblasts in the presence of active TGF-β1. However, treatment with molecules ChemB-737 and 334 led to significant reductions in αSMA, collagen I, Thy1, and periostin levels (Fig. 4).

**Fig. 5.**
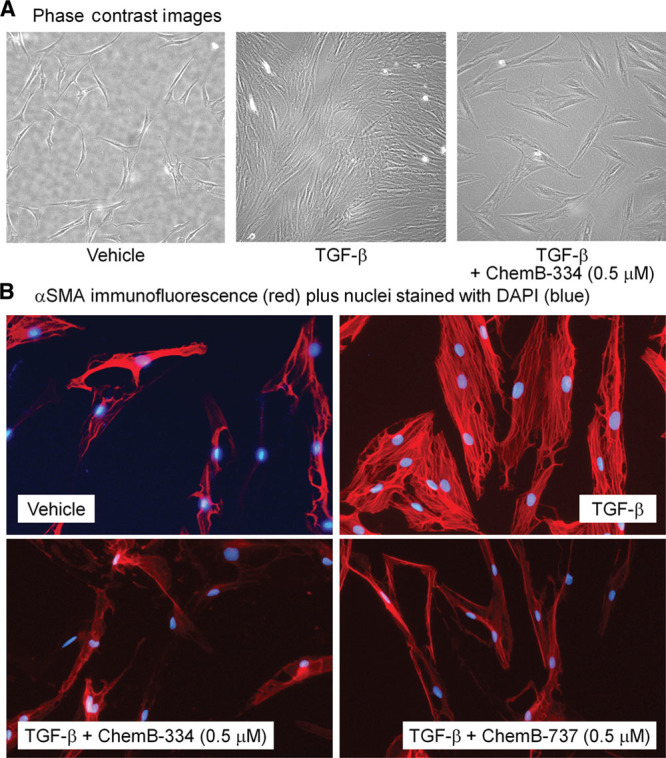
Capsular fibroblasts show morphologic differences between vehicle, TGF-β (2.5 ng/mL) only and TGF-β plus ChemB compounds. A, Bright-field microscopy images of vehicle, TGF or TGF + ChemB-334 (0.5 µM)-treated cells (*t* = 72 hours, 20× magnification). Vehicle cells, which did not receive either the TGF-β or treatment, show elongated shape, whereas TGF-β–treated cells show much broader shape with prominent intracellular fibers. TGF-β drives fibroblast proliferation, leading to much higher density of cells. Treating fibroblasts with both TGF-β and ChemB-334 prevents such cellular proliferation and myofibroblast morphologic changes, indicating that ChemB-334 can successfully prevent myofibroblast differentiation. B, Immunofluorescence images of fibroblasts show increased αSMA production in TGF-β–treated cells, a hallmark characteristic of myofibroblasts. Cells treated with ChemB-334 (0.5 µM) and 737 (0.5 µM) show elongated cells with much less prominent αSMA staining, indicating successful prevention of myofibroblast differentiation. DAPI, 4’,6-diamidino-2-phenylindole.

Cellular morphology was also analyzed, both with bright-field microscopy and immunofluorescence imaging (Fig. 5). Capsular fibroblasts displayed a clear myofibroblast morphologic change when treated with TGF-β1; the cells become much broader with prominent intracellular microfilaments, which are hallmark characteristics of myofibroblasts.^27^ However, ChemB-737– and ChemB-334–treated cells displayed morphologic features of nondifferentiated fibroblasts, rather than fully differentiated myofibroblasts. Furthermore, treated cells showed much weaker αSMA fluorescence compared with that of TGF-β1 only treated cells, indicating a significant reduction in αSMA production, hence reduced myofibroblast differentiation.

**Fig. 6.**
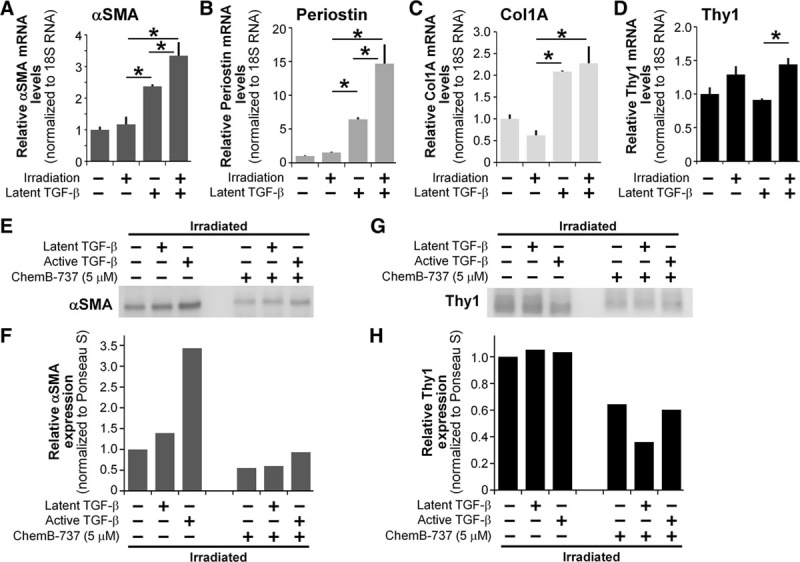
Ionizing radiation increases expression levels of myofibroblast markers. Quantitative real-time polymerase chain reaction results showing expression levels of αSMA (A), periostin (B), collagen 1A (C), and Thy1 (D), which are markers of capsular myofibroblasts. The effects of ChemB-737 compounds can be detected with or without exogenous TGF-β. Results indicate that ionizing radiation increases the TGF-β response in the capsular fibroblasts, which leads to increased myofibroblast differentiation and marker expression (compare bars 3 and 4 in A–D). The experiment was done in triplicate and significance was tested using one-way analysis of variance *P < 0.05. Cells treated with active TGF-β1 in the presence of radiation display more than 3 times the level of αSMA protein expression (E and F). Latent TGF-β also leads to an increased level of αSMA production, but to a lesser degree. Treating capsular fibroblasts with ChemB-737 reduces αSMA levels to near or below baseline, even in the presence of both latent and active TGF-β and ionizing radiation. In addition, ChemB-737 significantly reduces Thy1 expression in all conditions tested (G and H).

Ionizing irradiation is a known stimulus that can activate an array of cytokines including TGF-β, and especially TGF-β1 isoform.^28,29^ As described previously, increased levels of TGF-β can lead to increased myofibroblast formation and lead to pathologic fibrosis.^10^ Here, we used latent TGF-β1 and active TGF-β1 to test the effect of radiation of TGF-β activation. TGF-β1 can be activated by altering its state from latent to active forms. Stimuli such as an acidic environment, reactive oxygen species, and ionizing irradiation can lead to changes from latent to active TGF-β.^28–31^ To investigate the effects of these novel small molecules in an in vitro model of radiation, we irradiated the primary capsular fibroblasts with a 1-time dose of 10 Gy. ChemB-737 was selected for further evaluation in these tests based on results from Figures 1–5. Cells were treated with vehicle, latent, or active TGF-β1 plus or minus ChemB-737. After 5 days, samples were harvested for Western blot and RT-qPCR analysis. Cells that received latent TGF-β and irradiation showed a significant increase in mRNA levels of αSMA and periostin compared with that of nonirradiated cells (Fig. 6). Furthermore, an increase in Thy1 mRNA level was also noted with radiation. Cells that were treated with ChemB-737 showed significant reductions in levels of myofibroblast markers, in the presence of either latent or active TGF-β1 and ionizing irradiation (Fig. 6). Taken together, these results highlight the potential for ChemB-737 and potentially other molecules identified herein as novel anti-TGF-β and anti-capsular contracture agents.

## DISCUSSION

Radiation therapy can be an important part of breast cancer treatment. Postmastectomy radiation is effective in reducing breast cancer recurrence in qualifying patient populations.^32,33^ Unfortunately, radiation therapy can adversely affect the breast and local tissue and the healing process. One challenge is radiation-induced capsular contracture, as radiation therapy can significantly increase capsular contracture severity and incidence.^34^ Invasive capsulotomy and capsulectomy remain as the definitive treatments. Furthermore, capsular contracture tends to recur even with surgeries,^35^ subjecting patients to additional morbidity. Providing a noninvasive, preventative treatment to these patients who have already underwent cancer diagnosis and mastectomies would immensely benefit their physical and mental health.

Although the complete and exact mechanism of radiation-induced capsular contracture is unclear, one pathway involved is likely TGF-β activation by ionizing radiation. Here, we demonstrate that primary human capsular fibroblasts express significantly increased myofibroblast markers in the presence of latent TGF-β1 with ionizing irradiation (Fig. 6A–D). Increases may be explained by the activation of latent TGF-β via ionizing radiation, leading to increased myofibroblast differentiation. Importantly, cells that were treated with ChemB-737 showed decreased myofibroblast marker protein levels (Fig. 6E–H). This suggests that this novel TGF-β inhibitor may be an effective antiscarring agent even in the presence of ionizing radiation in vitro. The effect was seen with a single dose of 10 Gy radiation following 5 days of incubation postradiation. Although this dose regimen and timing is certainly different than adjuvant radiation given to breast cancer patients, it was chosen as an in vitro proof-of-concept dose considering the balance of cellular response to radiation, experimental limitations, and time constraints. In addition, previous studies with a mouse model of capsular contracture demonstrated effective capsule formation after 1 time dose of 10 Gy radiation.^36^ Considering that an average total dose a patient receives for breast cancer adjuvant radiation therapy is 45–50 Gy over a 4-week period,^37^ it is possible that the effect of ionizing radiation on TGF-β activation is greater or altogether different in patients. Future studies aimed at testing various radiation regimens will be helpful in understanding this further.

ChemB-282, 334, 737, and 671 are very similar structurally, with the only differences being the presence of halogen(s) (Fig. 2). Interestingly, the presence of halogens increases bioactivity as molecule ChemB-282 appears to be the least potent while ChemB-671 the most potent. Knowing the exact structures of these small molecules, future effort can be directed toward modification of their structures to increase the desired anti-TGF-β activity and increasing favorable pharmacokinetics properties; such optimization will increase the likelihood of clinical translation into therapies.

One of the proteins investigated was Thy1, which is a cell surface protein that plays an important role in myofibroblast differentiation. Previous studies by Koumas et al.^38^ showed that Thy1 (+) fibroblasts were capable of differentiating into myofibroblast upon TGF-β treatment, whereas Thy (−) cells failed to do so. It was also found that Thy1 expression is significantly elevated in irradiated capsular tissue.^25^ Here, these ChemB-737 family of molecules reduced Thy1 levels (at both RNA and protein), suggesting another possible mechanism of action in addition to the anti-TGF-β pathway.

Interestingly, TGF-β1 levels are often elevated in cancer patients, even before radiation, likely due to the malignancy itself.^39,40^ Reducing active TGF-β1 levels may not only help reduce soft tissue complication but also tumor progression and metastasis.^41^ Fibroblasts, or cancer-associated fibroblasts, are known to play a role in promotion of cancer, including breast cancer. TGF-β plays an important role in this process and activates cancer-associated fibroblasts, leading to tumor promotion.^42,43^ Suppression of myofibroblast activities may further benefit breast cancer survivors. Furthermore, there have been some studies looking at TGF-β1 gene polymorphisms and their potential role in different disease processes, such as radiation pneumonitis.^44^ Although there have been no studies focusing on TGF-β gene polymorphisms and capsular contracture, it may certainly play a role in the development of this pathologic fibrosis. Further investigation into this possibility is warranted.

The small molecules identified herein may lead to the development of an effective capsular contracture therapy, especially for those who receive adjuvant radiation therapy. However, before they can be considered for therapeutic options, more work must be done. Proper pharmacokinetics, toxicity studies, and delivery methods must be considered. Such development will require additional in vivo studies.^36^ Because these agents aim to prevent excessive myofibroblast differentiation and therefore capsule formation, they will likely be most effective if applied simultaneously or before radiation. The best drug delivery method may be direct application to the implant material to induce local, antifibrotic effects that can target the root cause of radiation-induced capsular contracture with minimal undesirable systemic effects.

Although this study demonstrates a promising antimyofibroblast effect of novel anti-TGF-β agents, there are some limitations. These proof-of-principle experiments were done in vitro in primary human capsular fibroblasts; however, additional in vivo studies will be important. Future studies using these compounds should include animal models utilizing the established radiation-induced psular contracture mouse model.^36^ Secondly, our study specifically tested TGF-β1 and not other isoforms. We focused on TGF-β1 as previous studies have demonstrated increased levels of active TGF-β1 with radiation, and its powerful proscarring and promyofibroblast effect.^45^ Finally, capsular contracture is a complex, multifaceted disease process. Although fibroblasts, myofibroblasts, ionizing radiation, and TGF-β all play a significant role disease development, there are likely multiple other factors such as cell type, foreign body response, immune cell–resident cell interactions, and other biological pathways that contribute to the pathology of capsular contracture. How these small molecule inhibitors of TGF-β alter these pathways remains unclear and requires investigation.

## CONCLUSIONS

Capsular contracture is a painful and disfiguring complication that occurs in patients undergoing implant-based breast reconstruction. Adjuvant radiation therapy greatly increases the incidence of capsular contracture. Radiation-induced capsular contracture is thought to be driven by TGF-β activation. Scar-forming and contracture causing myofibroblasts derive from capsular fibroblasts exposed to high levels of active TGF-β. Several novel small molecules identified from an anti-TGF-β screen effectively reduced capsular fibroblast to myofibroblast differentiation even in the presence of ionizing radiation. These results highlight novel therapeutic options that may be utilized in the future to prevent radiation-induced capsular contracture.
